# Association of pesticide exposure with respiratory health outcomes and rhinitis in avocado farmworkers from Michoacán, Mexico

**DOI:** 10.1016/j.scitotenv.2024.173855

**Published:** 2024-06-12

**Authors:** Cecilia S. Alcalá, Cynthia Armendáriz-Arnez, Ana M. Mora, Maria G. Rodriguez-Zamora, Asa Bradman, Samuel Fuhrimann, Christian Lindh, María José Rosa

**Affiliations:** a Department of Environmental Medicine and Public Health, Icahn School of Medicine at Mount Sinai, New York, NY, United States; b Escuela Nacional de Estudios Superiores (ENES) Unidad Morelia, Universidad Nacional Autónoma de México (UNAM), Michoacán, Mexico; c Center for Environmental Research and Community Health (CERCH), School of Public Health, University of California, Berkeley, CA, United States; d Escuela de Ingeniería en Seguridad Laboral e Higiene Ambiental (EISLHA), Instituto Tecnológico de Costa Rica, Cartago, Costa Rica; e Department of Public Health, University of California, Merced, CA, United States; f Department of Epidemiology and Public Health, Swiss Tropical and Public Health Institute (Swiss TPH), Allschwil, Switzerland; g University of Basel, Basel, Switzerland; h Division of Occupational and Environmental Medicine, Lund University, Lund, Sweden

**Keywords:** Pesticide, Mixtures, Respiratory health, Asthma, Wheeze, Occupational health

## Abstract

**Background:**

A growing literature suggests associations between occupational pesticide exposure and respiratory health. In this study, we aimed to examine the association of exposure to insecticides, fungicides, and herbicides, individually and as a mixture, with respiratory health outcomes and rhinitis in avocado farmworkers from Michoacán, Mexico.

**Material and methods:**

We conducted a cross-sectional study of 105 avocado farmworkers between May and August 2021. We quantified 12 insecticide, fungicide, and herbicide metabolites in urine samples collected during two study visits (8–10 weeks apart). We collected survey data on self-reported pesticide use during the 12 months prior to the baseline survey and estimated annual exposure-intensity scores (EIS) using a semi-quantitative exposure algorithm. We also assessed respiratory symptoms, including wheezing, chest tightness, wheezing after exercise, and night cough. We used generalized linear regression models to examine associations of individual urinary metabolite concentrations and annual EIS with respiratory health outcomes and rhinitis. Mixture effects were assessed using Bayesian Weighted Quantile Sum (BWQS) regression.

**Results:**

After adjusting for multiple comparisons, we observed mostly null associations of individual pesticide metabolite concentrations and annual EIS with the outcomes of interest. However, in BWQS analyses, we found evidence of a mixture association of urinary pesticide metabolites with increased odds of night cough (OR: 5.34, 95 % CrI: 1.67, 20.62). Pyrethroid metabolites 3-phenoxybenzoic acid and cis- and trans-3-(2,2-dichlorovinyl)-2,2-dimethylcyclopropanecarboxylic acid were the main contributors to this association (43 %).

**Conclusions:**

Our findings indicate that exposure to a mixture of pesticides, particularly pyrethroid insecticides, may be associated with night cough in avocado farmworkers.

## Graphical Abstract

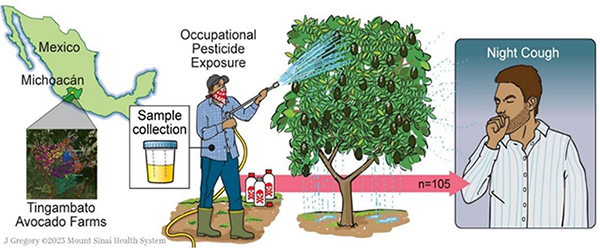

## 1. Introduction

Mexico is the world’s largest producer of avocados and accounts for approximately 45 % of global production (Gordean, 2021; SAGARPA, 2017). About 72 % of the avocados produced in Mexico are grown in 39 of the 113 municipalities in the state of Michoacan, which includes the municipality of Tingambato (Servicio de Información Agroalimentaria y Pesquera, 2023). Avocado production in Mexico has more than doubled in the last decade due to increasing demand, which has resulted in more widespread pesticide use (Bernabé, 2018; Denvir et al., 2021; Servicio de Información Agroalimentaria y Pesquera, 2023). S1 displays avocado farmworkers spraying pesticides in Michoacán, Mexico.

Farmworkers are at high risk of occupational exposure to pesticides through poor handling procedures during pesticide mixing and application (Baldi et al., 2012; Maroni et al., 2006). Additional exposure can occur due to fugitive emissions from neighboring orchards, re-entry into treated fields, during harvesting and packaging of produce, and from food and beverage consumption during pesticide application periods (Damalas and Koutroubas, 2016; Madrigal et al., 2023). Routes of occupational pesticide exposure in agricultural settings include inhalation, ingestion, and dermal absorption (Damalas and Koutroubas, 2016).

Multiple studies have reported associations between exposure to pesticides and a higher prevalence of respiratory and allergic outcomes (Ohlander et al., 2020). However, most studies have focused on organophosphate (OP) and carbamate pesticides. For instance, self-reported exposure to OP pesticides (e.g., parathion, malathion, chlorpyrifos) was associated with increased odds of wheeze among commercial pesticide applicators in Iowa and North Carolina (Hoppin et al., 2002). Similarly, exposure to OP pesticides (measured using erythrocyte acetylcholinesterase (AChE) activity levels), was associated with increased respiratory symptoms in farmworkers from Kenya (Ohayo-Mitoko et al., 2000) and India (Chakraborty et al., 2009; Chitra et al., 2006) and with reduced lung function in farmworkers from Spain (Hernández et al., 2008) and smallholder farmers in Uganda (Hansen et al., 2021). In Latin America and the Caribbean region, exposure to OP insecticides chlorpyrifos and terbufos was associated with increased odds of wheeze and shortness of breath among Indigenous women working in banana plantations in Costa Rica (Fieten et al., 2009). In addition, chronic exposure to pesticide mixtures was associated with allergic rhinitis and asthma in Colombian farmworkers (Díaz-Criollo et al., 2020).

Occupational studies that have evaluated the respiratory effects of exposure to classes other than OP pesticides have yielded mixed results (Sapbamrer and Seesen, 2020). For example, a study in an agricultural community in Thailand found null associations of self-reported pesticide exposure to pyrethroid insecticides, herbicides, and fungicides with respiratory symptoms and impaired lung function (Ratanachina et al., 2021). A small study of family farmers from Brazil observed that exposure to multiple pesticide classes and frequency of pesticide manipulation were associated with impaired lung function outcomes (Buralli et al., 2018). Similarly, another study of Brazilian farmworkers observed an association of self-reported pesticide mixing and application with increased odds of asthma-related respiratory symptoms but not with any specific pesticide classes (Faria et al., 2005). A large analysis from the U.S. Agricultural Health Study (AHS) found that self-reported use of pyrethroid insecticides and herbicides was associated with higher odds of allergic and non-allergic wheeze in male farmers (Hoppin et al., 2017). In an analysis of female farmers from the AHS, self-reported use of insecticides and several herbicides and fungicides was associated with atopic asthma, whereas only self-reported permethrin use was associated with non-atopic asthma (Hoppin et al., 2008). A higher self-reported lifetime exposure to insecticides, particularly carbamates, organochlorines, and pyrethroids, was associated with higher odds of doctor-diagnosed chronic bronchitis among male and female farmers from the AHS (Hoppin et al., 2007a). Notably, a study of rural women in South Africa did not find consistent associations between urinary pyrethroid metabolite concentrations and asthma-related outcomes (Mwanga et al., 2016). Likewise, higher concentrations of organochlorines but not of pyrethroid metabolites in urine were associated with a higher prevalence of respiratory symptoms in Ghanaian vegetable farmers (Quansah et al., 2016).

Risk assessment and regulation of pesticide exposure focus on single pesticides or within a single class, such as OP pesticides. This approach may fail to account for exposures to pesticide mixtures occurring in real-world situations (Guo et al., 2020; Lazarevic et al., 2019). Because pesticides may act on overlapping biological pathways and growing evidence suggests that joint pesticide exposure can be synergistically toxic (Nicolopoulou-Stamati et al., 2016), it is necessary to evaluate exposures to pesticide mixtures to understand their cumulative effects comprehensively (Claus Henn et al., 2014). In this study, we leveraged data from a cross-sectional study of avocado farmworkers in Michoacán, Mexico, to examine the association of occupational exposure to pesticides, both individually and as a mixture, with respiratory health outcomes and rhinitis.

## 2. Methods

### 2.1. Study population and procedures

From May 17 to 29, 2021, we enrolled 400 avocado farmworkers living in Tingambato, a rural community in the state of Michoacán, Mexico. This study was conducted in partnership with the Tingambato Local Plant Health Board (LPHB) and was advertised through local community groups and growers, on local radio, and via flyers posted around town as previously described (Rosa et al., 2024). Eligibility criteria included: ≥18 years of age, not pregnant, and had worked at an avocado farm registered at the Tingambato LPHB within the previous two weeks.

At the enrollment/baseline study visit, sociodemographic, medical, and occupational information was collected using a computer-based questionnaire in Spanish. Data on the frequency of use of the 12 pesticide active ingredients most commonly used in the study area (Castañeda-Ochoa et al., 2019) during the previous 12 months, as well as the frequency of personal protective equipment (PPE) use and hygienic behaviors after pesticide use (i.e., changing clothes, showering, sharing food and beverages consumption during pesticide application, washing and rinsing of pesticide tools or equipment) were also collected. Participants provided a urine sample after completing the questionnaire.

All farmworkers who participated in the baseline visit were invited to participate in a follow-up visit. However, only the first 105 (26.3 %) farmworkers who contacted the Tingambato LPHB or our research staff to express their interest in participating were enrolled in this second visit (conducted from August 17 to 27, 2021). The follow-up visit included the administration of a detailed questionnaire on respiratory health outcomes and rhinitis and the collection of a second urine sample. Both baseline and follow-up visits were conducted in person at the Tingambato LPHB offices. The study was approved by the Institutional Review Boards at UC Berkeley and Escuela Nacional de Estudios Superiores Unidad Morelia, Universidad Nacional Autónoma de México. All participants provided written informed consent at enrollment.

### 2.2. Exposure intensity scores (EIS)

We used a semi-quantitative exposure algorithm to estimate annual exposure intensity scores (EIS) during the 12 months prior to enrollment by combining information on application and mixing practices, frequency of PPE use, and hygienic behavior as previously described in detail (Rosa et al., 2024). Briefly, exposure-intensity scores were estimated using five factors: (1) mixing of pesticide active ingredients (MIX; yes/no, score 5 or 0), (2) applying pesticides outdoors using manual handheld backpack sprayers (APPLICATION; yes/no, score 8 or 0), (3) a PPE score that considered coverage of different body areas and frequency of use (PPE, scores ranged from 0.14 to 1), (4) time between pesticide application and change of clothes (CHANGE, scores ranged from 0.7 to 1), and (5) time between pesticide application and showering (SHOWER, scores ranged from 0.7 to 1).


EIS=(MIX+APPLICATION)×PPE×CHANGE×SHOWER

Annual EIS scores were calculated by multiplying the EIS by the frequency (days/month) of mixing or applying any of the 12 active pesticide ingredients listed in Table S1 in the last 12 months.


ANNUALEIS=EIS×FREQUENCYOFPESTICIDEEXPOSUREINTHEPAST12MONTHS

### 2.3. Urinary pesticide metabolite assessment

Spot urine samples were collected at baseline and follow-up visits after handwashing and stored in 100 mL single-use sterile polypropylene containers (Vacuette^®^, sterile) at 4 °C until the end of the fieldwork day. Study staff aliquoted samples into 15-mL test tubes (PerformRTM Centrifuge tubes, Labcon^®^, sterile) and stored them at −80 °C until shipment in dry ice to Lund University, Sweden, for analysis.

Urine samples were analyzed for 12 pesticide biomarkers (Table 1), including four OP insecticide metabolites [2-isopropyl-4-methyl-6-hydroxypyrimidine (IMPy, a metabolite of diazinon), 3,5,6-trichloro-2-pyridinol (TCPy, a metabolite of chlorpyrifos), 4-bromo-2-chlorophenol (BCP, metabolite of profenophos), and malathion diacid (MDA, metabolite of malathion)]; three metabolites of pyrethroid insecticides [3-phenoxybenzoic acid (3-PBA), cis- and trans-3-(2,2-dichlorovinyl)-2,2-dimethylcyclopropanecarboxylic acid (DCCA)]; four fungicide metabolites [hydroxy-pyrimethanil (OH-P, a metabolite of pyrimethanil), hydroxy-boscalid (OH-BOS, a metabolite of boscalid), hydroxy-thiabendazole (OH-T, a metabolite of thiabendazole), and hydroxy-tebuconazole (OH-TEB, a metabolite of tebuconazole)]; and one herbicide [2,4-dichlorophenoxyacetic acid (2,4-D; parent compound)].

Urine samples were analyzed using methods previously described (Mora et al., 2022; Norén et al., 2020). In brief, samples were de-conjugated using β-glucuronidase/arylsulfatase and diluted. Samples were then analyzed using liquid chromatography-triple quadrupole linear ion trap mass spectrometry (LC/MS/MS; QTRAP 5500 or 6500 +; AB Sciex, Framingham, MA, USA). All batches included in-house quality control samples (QC) and laboratory blanks, and 30 % of the samples were analyzed in duplicates. Between-run precisions of QC samplers were 3–25 %. The limits of detection (LOD) and detection frequencies are shown in Table 1. The laboratory regularly participates in the G-EQUAS quality assessment scheme for the analysis of TCP and 3-PBA, organized by the German Society of Occupational Medicine.

Urinary specific gravity (kg/L) was measured using a digital refractometer (Refractometer 30PX, Mettler Toledo, Columbus, OH, USA) (Rosa et al., 2024). Metabolite concentrations were normalized for dilution using the formula $P_{SG}=P\times [(1.016-1)/(SG-1)]$, where $P_{SG}$ is the specific gravity-corrected pesticide biomarker concentration (μg/L), P is the observed pesticide biomarker concentration (μg/L), *SG* is the specific gravity of the urine sample (determined using a hand refractometer), and 1.016 kg/L is the average specific gravity for our study sample (Rosa et al., 2024). We included in our analyses pesticide metabolites with detection frequencies of 50 % or more at both study visits (Lubin et al., 2004). Machine read values were used when samples were below the LOD.

### 2.4. Respiratory outcomes and rhinitis assessment

At the follow-up visit, participants were administered a short version of the Spanish European Community Respiratory Health Survey (ECRHS) questionnaire (Burney et al., 1994), which has been previously used in studies of Latin American populations (Fieten et al., 2009), to identify symptoms of asthma, chronic bronchitis, rhinitis, and eczema in the last 12 months.

We derived an asthma symptom scale which consisted of a simple sum of positive answers to five respiratory symptoms (score range of 0–5): (i) wheezing in the last 12 months (current wheeze), (ii) woken with a feeling of chest tightness in last 12 months (chest tightness), (iii) shortness of breath at rest in last 12 months (shortness of breath), (iv) shortness of breath after exercise in last 12 months (wheeze after exercise), and (v) woken by shortness of breath in last 12 months (shortness of breath at night) (Sunyer et al., 2007). Due to the distribution of the symptom score, scores with >1 symptom were collapsed into a single category. We also examined the following symptoms individually: current wheeze, chest tightness, and wheeze after exercise. Shortness of breath was not examined individually due to low prevalence. Lastly, we examined reports of waking up in the past 12 months from coughing (night cough) and reports of sneezing or a runny or blocked nose without having a cold or the flu during the last 12 months (current rhinitis).

### 2.5. Statistical analysis

We averaged pesticide concentrations across the two urine samples collected for each farmworker and log_2_-transformed these average concentrations to reduce the influence of extreme values (if a participant provided only one urine sample (*n* = 8), we used that measurement). We used generalized linear models with a binomial distribution to examine the association of pesticide exposure with respiratory health outcomes and rhinitis among farmworkers. We utilized Poisson regression for the asthma symptom score models. We adjusted our models for age (continuous) and smoking history (categorical: never, former, or current smoker). The estimated adjusted odds ratio (OR) or incidence rate ratio (IRR; for asthma symptom score) represent the change in the odds of an outcome or in the risk of a higher asthma score per two-fold increase in concentrations of each urinary pesticide metabolite after adjusting for potential confounders. All individual analyses were adjusted for the false discovery rate (FDR) to account for multiple comparisons.

For mixtures analyses, we used Bayesian Weighted Quantile Sum (BWQS) regression (Colicino et al., 2020) to evaluate the association of a pesticide mixture with each respiratory health outcome and rhinitis among farmworkers. Each BWQS model utilized an uninformative prior with a normal distribution and a single chain comprising of 10,000 iterations, with 5000 warm-up and a thinning parameter of 10. All models were checked for good convergence by checking the potential scale-reduction statistic, $\hat{R}$ was approximately equal to 1. To assess evidence of the associations, we present 95 % credible intervals (CrI).

In sensitivity analyses, we additionally adjusted for 1) wood stove use (dichotomous: yes/no), 2) animal exposure in the home (defined as being exposed to a dog, cat, turtle, or any other pet in the home or to horses, cows, pigs, sheep, goats, chickens, rabbits or any other animal in the home or property where they lived), 3) educational level (dichotomous: ≤6th grade or > 6th grade), and we excluded women from all models because respiratory health and rhinitis outcomes are known to vary by sex (Pinkerton et al., 2015) (we were underpowered to examine the associations of interest only among women). We conducted all analyses using R Version 4.1.1 (R Development Core Team).

## 3. Results

### 3.1. Study population characteristics

A total of 105 farmworkers were included in this study. Participants had a mean (SD) age of 40.4 (14.9) years (Table 2). Most farmworkers were male (96.2 %), had completed 6th grade (69.5 %), and about half of them never smoked (51.4 %). Most participants had a history of pesticide application at work (90.5 %) and reported applying pesticides in the last 12 months (79.0 %). We did not find significant differences in baseline characteristics between those included in the follow-up visit (*n* = 105) and those excluded (first visit only, *n* = 295).

The most prevalent respiratory symptoms in the last 12 months were night cough and exercise wheeze (15.2 % both) (Table 3). About 10 % of farmworkers reported having more than one asthma symptom, whereas almost one-fifth (18.1 %) reported current rhinitis. One study participant reported chronic bronchitis symptoms (defined as experiencing a productive cough most days for at least 3 months a year); three reported having (ever) had asthma (defined as the individual responding to the question “Have you ever had asthma in your life?”); no one reported active asthma (defined as the person experiencing asthma symptoms during or after physical activity); one reported physician-diagnosed asthma, and five reported current eczema (experiencing eczema symptoms in the past 12 months).

Detection frequencies for the 12 pesticide metabolites ranged from 15 % to 100 %, with nine of the metabolites having a detection frequency of 50 % or more in urine samples collected at both study visits (Table 1). Geometric mean (GM) [geometric standard deviation (GSD)] specific gravity-adjusted urinary 3-PBA, and cis- and trans-DCCA concentrations averaged over the two study visits were 1.8 ng/mL (2.0), 1.8 ng/mL (1.8), and 1.2 ng/mL (1.7), respectively (Table 1). Correlations between averaged urinary pesticide metabolites varied widely (r_s_ = −0.22 to 0.77) but were the highest between the two DCCA enantiomers (r_s_ = 0.77) and between both enantiomers and 3-PBA (r_s_ = 0.70 and 0.62 with cis- and trans-DCCA, respectively) (Rosa et al., 2024).

### 3.2. Results from individual pesticide metabolite regression models

The associations of individual urinary concentrations of pesticide metabolites with respiratory health outcomes and rhinitis among farmworkers are shown in Table 4. Higher EIS scores were associated with increased odds of night cough (OR per unit increase in scores: 1.02, 95 % CI: 1.00, 1.04). Higher concentrations of all three pyrethroid metabolites (i.e., 3-PBA, cis- and trans-DCCA) were also associated with increased odds of night cough (OR per two-fold increase in urinary concentrations: 2.04, 95 % CI: 1.17, 3.76; OR: 2.52, 95 % CI: 1.33, 5.25; OR: 2.56, 95 % CI: 1.18, 6.02; respectively). Notably, all these associations became null after adjusting for multiple comparisons using FDR correction.

### 3.3. Results from the pesticide mixture effects models

**Fig. 1.**
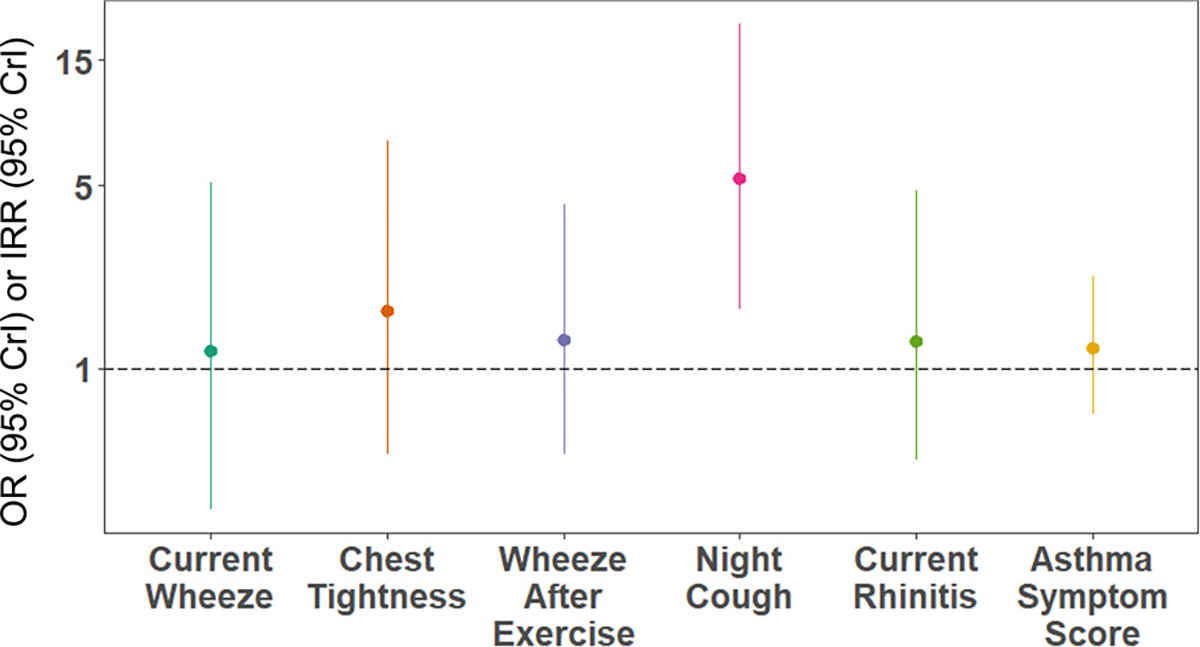
Association of occupational exposure to pesticide mixtures with respiratory health outcomes and rhinitis among avocado farmworkers in Michoacan, Mexico (n = 105). Odds ratio (OR) or Incidence rate ratio (IRR) and 95 % credible interval (CrI).

**Fig. 2.**
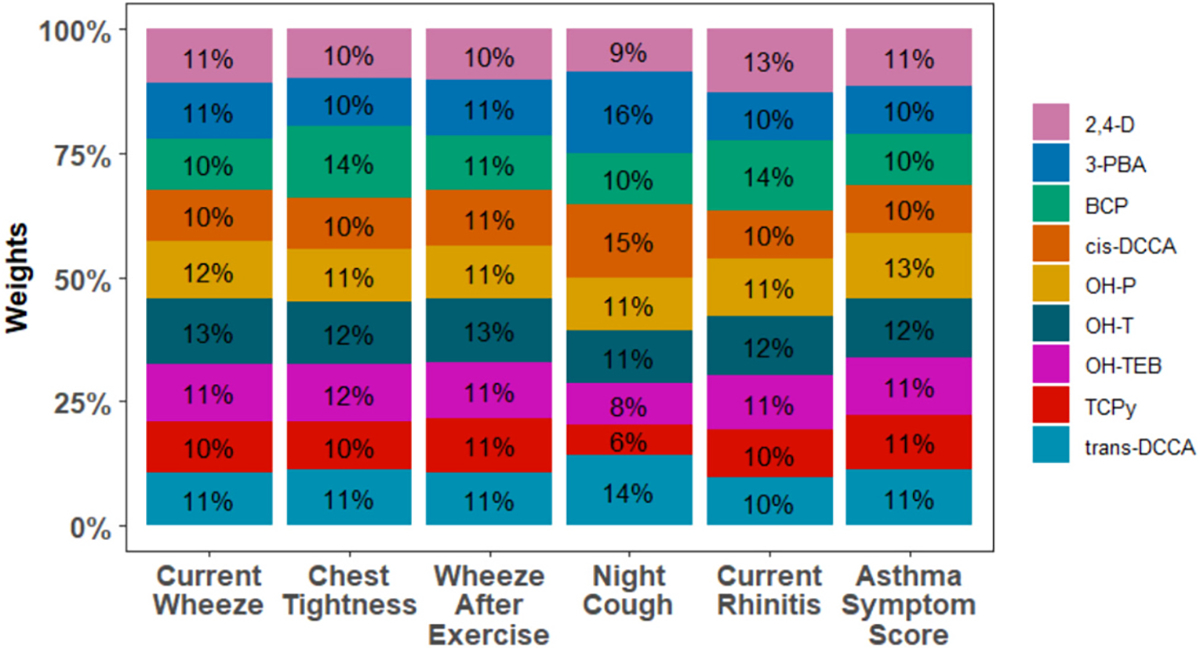
Contribution of each urinary pesticide metabolite to the mixture for respiratory and rhinitis outcomes assessed in avocado farmworkers, Michoacán, Mexico. May–August 2021.

We observed a positive association between the urinary metabolite mixture and increased odds of night cough (OR: 5.34, 95 % CrI: 1.67, 20.62; Fig. 1 and Table S2). Pyrethroids were the largest contributors to the mixture effect (41 %) (Fig. 2). We did not observe associations of the pesticide mixture with other respiratory health outcomes or rhinitis.

### 3.4. Sensitivity analyses

The associations between urinary pyrethroid metabolites and increased odds of night cough remained unchanged after adjustment for wood stove use, animal exposure, and education and after excluding women from our models (Table S3). However, associations between annual EIS and night cough were somewhat attenuated after adjusting for animal exposure and excluding women from the models (Table S3).

## 4. Discussion

In this study of avocado farmworkers in Michoacán, Mexico, we observed mostly null associations of individual pesticide metabolite concentrations and annual EIS with respiratory health outcomes and rhinitis, after adjusting for multiple comparisons. However, in BWQS analyses, we observed an association between the pesticide metabolite mixture and increased odds of night cough. The pyrethroid metabolites 3-PBA, cis- and trans-DCCA were identified as the largest contributors to the mixture association.

Several studies have examined the association between pyrethroid exposure and respiratory outcomes in occupationally exposed populations with mixed results (Hoppin et al., 2008; Hoppin et al., 2017; Hoppin et al., 2007b; Hudson et al., 2014; Islam et al., 2022; Mwanga et al., 2016; Ratanachina et al., 2021). For instance, a survey of pyrethrin- and pyrethroid-related illnesses and injuries in the United States (2000–2008) found an increased prevalence of acute respiratory effects, including wheeze, dyspnea, and cough (Hudson et al., 2014). In an analysis of male farmers from the AHS, exposure to the pyrethroids permethrin and pyrethrins was associated with higher odds of both allergic and non-allergic wheeze (Hoppin et al., 2017). In an analysis of female farmers from the AHS, self-reported permethrin use for crops and for animals was associated with higher odds of atopic and non-atopic asthma, respectively (Hoppin et al., 2008). Likewise, a higher self-reported lifetime exposure to pyrethroids was associated with higher odds of doctor-diagnosed chronic bronchitis among male and female farmers from the AHS (Hoppin et al., 2007a). Conversely, a cross-sectional study of farmworkers and non-farmworkers in Thailand observed null associations of reported use of pyrethroids with chronic cough or lung function outcomes (Ratanachina et al., 2021). A study of farmworker and non-farmworker women in South Africa found null associations between urinary pyrethroid metabolites with serum cytokine patterns and asthma-related outcomes, including doctor-diagnosed asthma and allergic sensitization (Mwanga et al., 2016). A study of farmworkers from Ghana also reported null associations of exposure to pyrethroids with chronic cough, phlegm, and wheeze (Quansah et al., 2016). Notably, studies of pediatric populations have reported associations between pyrethroid exposure and cough (Elsiwi et al., 2024; Islam et al., 2023). In a cross-sectional study, the sum of urinary pyrethroid metabolite concentrations was associated with elevated odds of current cough in 5-year-old Costa Rican children (Islam et al., 2023), and maternal urinary concentrations of pyrethroid metabolites measured at delivery were associated with higher risk of night cough in children aged 5 years in a South African birth cohort (Elsiwi et al., 2024).

Animal and in vitro studies have shown that pesticides can impact the respiratory system through a variety of mechanisms including, but not limited to, irritation, generation of oxidative stress, and inflammation (Dar et al., 2019; Ileriturk et al., 2022; Kumar et al., 2023; Şekeroğlu et al., 2020). For example, exposure to the pyrethroid insecticide cypermethrin was associated with higher lung toxicity, including higher levels of reactive oxygen species (ROS) and inflammatory markers in rats (Ileriturk et al., 2022). Another study in rats reported that exposure to the pyrethroid insecticide bifenthrin was associated with lung tissue lipid peroxidation and alteration of antioxidant enzymes (Dar et al., 2019). Lastly, an in vitro study of human lung fibroblast cells showed that exposure to both deltamethrin and thiacloprid was associated with higher levels of ROS production (Şekeroğlu et al., 2020).

Strengths of our study include 1) comprehensive exposure assessment utilizing both estimates for long-term (i.e., annual EIS) and short-term (i.e., repeated pesticide biomarker measurements) exposure, 2) the administration of a validated questionnaire to assess respiratory health and rhinitis outcomes, and 3) the use of BWQS to assess mixture effects, account for co-exposures, and identify key exposures. Nevertheless, our study also has limitations, including the small sample size and cross-sectional design. It is possible that some associations in this cross-sectional analysis were biased due to a healthy worker effect. Furthermore, since symptoms and outcomes were assessed via questionnaire (i.e., we did not conduct clinical assessments such as lung function tests or skin prick tests, nor measure specific or total immunoglobulin E), the potential for reporting or recall bias among farmworkers cannot be ruled out. It is important to highlight that the pesticide biomarkers measured in our study are rapidly metabolized and may only reflect recent pesticide exposure (Barr, 2008). However, to minimize exposure measurement error and intra-individual variability, we utilized the average concentrations of two spot urine samples, which is a better predictor of long-term pesticide exposure than a single spot sample (Bradman et al., 2013; Perrier et al., 2016). Lastly, although we adjusted urinary pesticide metabolite concentrations for specific gravity to account for differences in dilution (i.e., hydration status), we cannot dismiss the impact of genetic and physiological factors such as exercise, fasting status, and kidney function on these concentrations (Cuomo et al., 2022; Gordon, 2003).

In this cross-sectional study of avocado farmworkers, we observed evidence of an association between a mixture of urinary pesticide metabolites and increased odds of night cough; pyrethroid metabolites were the strongest contributors to this association. Given that night cough can be caused by multiple medical conditions (e.g., asthma, chronic obstructive pulmonary disease, gastroesophageal reflux disease, postnasal drip), further investigation is warranted. Nevertheless, our findings highlight the need to assess the long-term respiratory effects of non-OP pesticides in occupational populations. These findings have important public health implications, considering the widespread use of pyrethroid pesticides in Latin American and Caribbean countries for agricultural and vector control purposes.

## Supplementary Material

Appendix A

## Figures and Tables

**Table 1:** Distribution of urinary pesticide metabolite (specific gravity-adjusted) concentrations (not imputed) measured in avocado farmworkers’ urine samples collected at one or two time points, Michoacán, Mexico (*n* = 105). May–August 2021.

Urinary biomarkers^a^	LOD	% > LOD		Average of two measurements					
	
		1st measurement^b^	2nd measurement^c^	GM (GSD)	Min	P25	P50	P75	Max

Organophosphates (insecticides)									
TCPy	0.10	100	100	1.88 (1.72)	0.63	1.40	1.80	2.43	10.9
IMPy	0.05	36	27	0.04 (2.97)	<LOD	<LOD	<LOD	0.07	1.27
MAD	0.10	36	34	0.12 (7.36)	<LOD	<LOD	<LOD	0.28	58.3
BCP	0.05	57	76	0.40 (4.80)	<LOD	0.13	0.53	1.17	7.91
Pyrethroids (insecticides)									
3-PBA	0.20	99	96	1.78 (2.00)	0.25	1.18	1.70	2.55	12.9
cis-DCCA	0.40	92	94	1.75 (1,84)	0.46	1.15	1.64	2.55	15.7
trans-DCCA	0.70	74	70	1.23 (1.68)	<LOD	0.88	1.26	1.61	6.75
Fungicides									
BOS-OH	0.50	18	15	0.20 (2.67)	<LOD	<LOD	<LOD	<LOD	1.71
OH-P	0.05	70	66	0.22 (6.74)	<LOD	0.06	0.14	0.33	208
OH-T	0.05	76	53	0.13 (4.83)	<LOD	0.05	0.12	0.32	30.7
OH-TEB	0.10	76	82	0.36 (2.24)	<LOD	0.22	0.34	0.58	4.17
Herbicides									
2,4-D	0.05	100	97	0.40 (2.53)	0.09	0.25	0.33	0.48	75.9

Abbreviations: BCP, 4-bromo-2-chlorophenol; 2,4-D, 2,4-dichlorophenoxyacetic acid; DCCA, 3-(2,2-dichlorovinyl)-2,2-dimethylcyclopropanecarboxylic acid; 3-PBA, 3-phenoxybenzoic acid; OH-P, 3-hydroxy-pyrimetanil; OH-T, 5-hydroxy-thiabendazole; TCPy, 3,5,6-trichloro-2-pyridinol; IMPy, 2 –isopropyl-4-methyl-6-hydroxypyrimidine; MAD, malathion diacid; BOS-OH, hydroxyl-boscalid; LOD, limit of detection; GM, geometric mean; GSD, geometric standard deviation; OH-TEB, hydroxy-tebuconazole.

a Units are ng/mL for all urinary pesticide biomarkers.

b Urine samples collected from 105 farmworkers.

c Urine samples collected from 97 farmworkers.

**Table 2:** Descriptive characteristics of overall sample and subsample of avocado workers from Michoacán, Mexico. May–August 2021.

Variable	All farmworkers (*n* = 400)	Included in follow-up visit (n = 105)	Excluded from follow-up visiting (n = 295)	*p*-value^a^

Age				
Mean (SD)	38.6 (13.8)	40.4 (14.9)	38.0 (13.4)	0.15
Missing, n (%)	3 (0.8)	1 (1.0)	2 (0.7)	
Sex, n (%)				0.38
Female	9 (2.2)	4 (3.8)	5 (1.7)	
Male	391 (97.8)	101 (96.2)	290 (98.3)	
Educational level, n (%)				0.98
≤6th grade	116 (68.0)	32 (30.5)	84 (28.5)	
>6th grade	272 (29.0)	73 (69.5)	199 (67.5)	
Missing	12 (3.0)	0 (0)	12 (4.1)	
Smoking history, n (%)				0.56
Never smoker	226 (56.5)	54 (51.4)	172 (58.3)	
Former smoker	87 (21.8)	25 (23.8)	62 (21.0)	
Current smoker	80 (20.0)	23 (21.9)	57 (19.3)	
Missing	7 (1.8)	3 (2.9)	4 (1.4)	
Wood stove use, n (%)				0.26
No	213 (53.3)	47 (44.8)	166 (56.3)	
Yes	147 (36.8)	41 (39.0)	106 (35.9)	
Missing	40 (10)	17 (16.2)	23 (7.8)	
Animal exposure,^b^ n (%)				
No	–	13 (12.4)	–	
Yes	–	87 (82.9)	–	
Missing	–	5 (4.8)	–	
Time working in agriculture (years)				0.68
Mean (SD)	21.8 (15.2)	22.4 (16.5)	21.6 (14.7)	
Missing, n (%)	6 (1.5)	3 (2.8)	3 (1.0)	
Ever applied pesticides, n (%)				0.67
No	37 (9.3)	8 (7.6)	29 (9.8)	
Yes	361 (90.3)	95 (90.5)	266 (90.2)	
Missing	2 (0.5)	2 (1.9)	0 (0)	
Time applying pesticides (years)				0.37
Mean (SD)	15.7 (12.8)	16.7 (11.7)	15.4 (13.2)	
Missing, n (%)	43 (10.8)	12 (11.4)	31 (10.5)	
Applied pesticides in last 12 months, n (%)				0.84
No	73 (18.1)	22 (21.0)	51 (17.4)	
Yes	325 (81.3)	83 (79.0)	242 (82.0)	
Missing	2 (0.6)	0 (0)	2 (0.6)	
Annual exposure intensity scores (EIS)				0.80
Mean (SD)	13.9 (25.1)	13.3 (23.4)	14.0 (25.8)	
Median [min-max]	0 [0, 152]	0 [0, 114]	0 [0, 152]	

a Differences between farmworkers included in the follow-up visit and those excluded from this visit (*t*-test for continuous variables and chi-square test for categorical variables).

b Variable only collected at follow-up. Animal exposure was defined as being exposed to a dog, cat, turtle, or any other pet in the home or to horses, cows, pigs, sheep, goats, chickens, rabbits, or any other animal in the home or property where they lived.

**Table 3:** Prevalence of respiratory health outcomes and rhinitis assessed in avocado workers from Michoacan, Mexico (n = 105). May–August 2021.

Outcome	n (%)

Wheeze	
No	95 (90.5)
Yes	10 (9.5)
Chest tightness	
No	93 (88.6)
Yes	12 (11.4)
Exercise wheeze	
No	89 (84.8)
Yes	16 (15.2)
Shortness of breath	
No	101 (96.2)
Yes	4 (3.8)
Shortness of breath at night	
No	100 (95.2)
Yes	5 (4.8)
Night cough	
No	89 (84.8)
Yes	16 (15.2)
Ever asthma	
No	102 (97.1 %)
Yes	3 (2.9 %
Doctor-diagnosed asthma	
No	104 (99.0 %)
Yes	1 (1.0 %)
Active asthma	
No	105 (100 %)
Asthma symptom score	
0	74 (70.5)
1	21 (20.0)
>1	10 (9.5)
Chronic Bronchitis	
Yes	1 (1.0 %)
Rhinitis	
No	85 (81.0)
Yes	19 (18.1)
Missing	1 (1.0)
Current eczema	
No	100 (95.2 %)
Yes	5 (4.8 %)

**Table 4:** Associations of urinary concentrations of pesticide metabolites (per two-fold increase in specific-gravity adjusted concentrations) and annual exposure intensity scores (per one-unit increase) with respiratory health outcomes and rhinitis among avocado farmworkers in Michoacan, Mexico. May–August 2021.

Pesticide class/metabolite	Current wheeze (n = 105)	Chest tightness (n = 105)	Exercise wheeze (n = 105)	Night cough (n = 105)	Current rhinitis (*n* = 104)	Asthma symptom score (n = 105)
						
	*OR (95 % CI)*	*OR (95 % CI)*	*OR (95 % CI)*	*OR (95 % CI)*	*OR (95 % CI)*	*IRR (95 % CI)*

Organophosphates (insecticides)						
TCPy	0.54 (0.19, 1.34)	0.98 (0.42, 2.13)	0.90 (0.43, 1.81)	1.00 (0.48, 1.99)	1.34 (0.71, 2.53)	0.96 (0.63, 1.42)
BCP	0.98 (0.75, 1.32)	1.30 (0.97, 1.83)	0.89 (0.71, 1.12)	0.98 (0.78, 1.25)	1.14 (0.91, 1.45)	0.98 (0.86, 1.12)
Pyrethroids (insecticides)						
3-PBA	0.90 (0.45, 1.71)	0.99 (0.53, 1.80)	1.29 (0.76, 2.20)	**2.04 (1.17, 3.76)**	0.86 (0.50, 1.43)	1.13 (0.84, 1.51)
cis-DCCA	1.18 (0.57, 2.39)	1.15 (0.57, 2.29)	1.55 (0.86, 2.89)	**2.52 (1.33, 5.25)**	0.82 (0.45, 1.43)	1.26 (0.90, 1.75)
trans-DCCA	1.35 (0.55, 3.30)	1.31 (0.55, 3.12)	1.22 (0.58, 2.57)	**2.56 (1.18, 6.02)**	0.64 (0.31, 1.27)	1.18 (0.77, 1.78)
Fungicides						
OH-P	0.95 (0.69, 1.21)	0.91 (0.66, 1.16)	0.90 (0.68, 1.12)	1.01 (0.81, 1.22)	1.00 (0.81, 1.21)	0.93 (0.80, 1.06)
OH-T	1.13 (0.86, 1.47)	1.10 (0.84, 1.43)	1.00 (0.79, 1.25)	1.07 (0.84, 1.34)	1.09 (0.88, 1.34)	1.07 (0.94, 1.21)
OH-TEB	0.81 (0.41, 1.43)	0.90 (0.49, 1.54)	0.91 (0.55, 1.46)	1.03 (0.64, 1.62)	0.67 (0.39, 1.06)	0.87 (0.65, 1.15)
Herbicides						
2,4-D	0.56 (0.22, 1.14)	1.05 (0.61, 1.55)	0.76 (0.39, 1.21)	0.74 (0.37, 1.19)	1.25 (0.88, 1.75)	0.84 (0.60, 1.10)
Annual EIS	1.01 (0.99, 1.04)	1.00 (0.96, 1.03)	0.99 (0.96, 1.02)	**1.02 (1.00, 1.04)**	0.98 (0.94, 1.00)	1.00 (0.98, 1.01)

*Abbreviations*: BCP, 4-bromo-2-chlorophenol; 2,4-D, 2,4-dichlorophenoxyacetic acid; DCCA, 3-(2,2-dichlorovinyl)-2,2-dimethylcyclopropanecarboxylic acid; EIS, exposure intensity scores; 3-PBA, 3-phenoxybenzoic acid; OH-P, 3-hydroxy-pyrimetanil; OH-T, 5-hydroxy-thiabendazole; OH-TEB, hydroxy-tebuconazole; TCPy, 3,5,6-trichloro-2-pyridinol. Bolded results have p-value <0.05 without FDR correction. All models were adjusted for age and smoking history.

## Data Availability

Data will be made available on request.

## HIGHLIGHTS

- First study to explore occupational exposure to pesticides and respiratory health outcomes among avocado farmworkers in Mexico.
- Avocado production in Mexico has more than doubled in the last decade due to increasing demand.
- Occupational exposure to a pesticide mixture may be associated with night cough among farmworkers.
