# Supplementary material for: Association of pesticide exposure with respiratory health outcomes and rhinitis in avocado farmworkers from Michoacán, Mexico
Source: Sci Total Environ. Author manuscript; Available in PMC 2024 Oct 1. (PMC11250725; doi:10.1016/j.scitotenv.2024.173855)
Supplement: Appendix A [file NIHMS2005271-supplement-Appendix_A.docx]

**SUPPLEMENTARY MATERIAL**

**S1.** Avocado farmworkers spraying pesticides in Michoacán, Mexico.

https://drive.google.com/file/d/12Yn4pa69mCciUoETABLhbX4TNq8T8Ubs/view

**Figure S1.** Directed Acyclic Graph (DAG) of assumed dependencies of occupational exposure to pesticides with respiratory health outcomes, rhinitis, and other health-related factors.


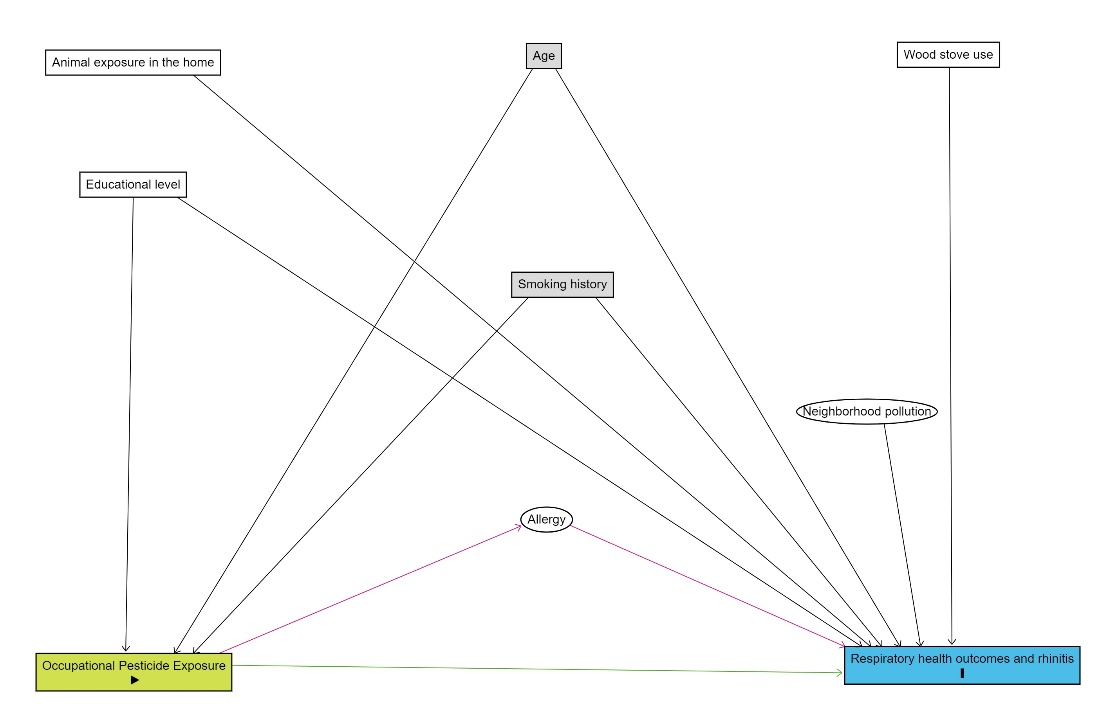


| **Table S1.** Description of pesticide active ingredients and commercial names assessed via questionnaire in this study of avocado farmworkers from Michoacan, Mexico (n=105). May-August 2021. | | | |
| --- | --- | --- | --- |
| **Pesticide active ingredient** | **Commercial name** | **Pesticide class** | **Pesticide group** |
| Abamectin | Agromectin | Avermectin | Insecticide and anthelmintic |
| Cypermethrin | Mustang max | Synthetic pyrethroid | Insecticide |
| Permethrin | Anatrin, Premier | Synthetic pyrethroid | Insecticide |
| Lambda cyhalothrin | Morgan, Lucambda, Manda | Synthetic pyrethroid | Insecticide |
| Imidacloprid | Manager, Uniprid | Neonicotinoid | Insecticide |
| Lambda cyhalothrin + Imidacloprid | Corax | Synthetic pyrethroid + neonicotinoid | Insecticide |
| Thiametoxam | Opta | Neonicotinoid | Insecticide |
| Malathion | Malathion 1000 | Organophosphate | Insecticide |
| Spinetoram | Exalt | Tetracyclic macrolide | Insecticide |
| Azoxystrobin | Axotron, Valgo | ß-methoxyacrylate | Fungicide |
| Thiabendazole | Zio, Tecto 60 | Benzimidazole | Fungicide |
| Glyphosate | Rudo, Glyphosate super, Velfosato | (non accepted) Glycine | Herbicide |
| Paraquat | Velquat, Sagacuat, Gramoxone | Viologen | Herbicide |

| **Table S2.** Association of occupational exposure to pesticide mixtures with respiratory health outcomes and rhinitis among avocado farmworkers in Michoacan, Mexico (n=105). May-August 2021. | | | | | | | | |
| --- | --- | --- | --- | --- | --- | --- | --- | --- |
| OR (95% CrI) | **Current wheeze** | **Chest tightness** | **Wheeze after exercise** | **Night cough** | **Current rhinitis** | **Asthma symptom score** | |  |
|  | 1.17 (0.29, 5.14) | 1.66 (0.47, 7.40) | 1.29 (0.47, 4.20) | **5.34 (1.67, 20.62)** | 1.27 (0.45, 4.78) | -- | |  |
| IRR (95% CrI) | -- | -- | -- | -- | -- | 1.20 (0.66, 2.24) | |  |
| *Abbreviations*: IRR, incidence rate ratio. CrI, credible interval. All models were adjusted for age and smoking history. | | | | | | |  |  |

| **Table S3.** Associations [OR (95% CI)] of urinary concentrations of pesticide metabolites (per two-fold increase in specific-gravity adjusted concentrations) and annual exposure intensity scores (per one-unit increase) with night cough, after adjusting by additional covariates or excluding subgroups, among avocado farmworkers in Michoacán, Mexico. May-August 2021. | | | | | | | | |
| --- | --- | --- | --- | --- | --- | --- | --- | --- |
| **Pesticide class/metabolite** | **Adjusting for wood stove use (n=105)** | **Adjusting for education (n=105)** | | **Adjusting for exposure to animals (n=105)** | | **Excluding women (n=101)** | |  |
| Organophosphates (insecticides) | | |  | |  | |  | |
| TCPy | 1.00 (0.48, 1.99) | 0.99 (0.48, 1.97) | | 1.00 (0.48, 2.00) | | 1.02 (0.50, 2.01) | |  |
| BCP | 0.98 (0.78, 1.25) | 0.98 (0.78, 1.25) | | 0.98 (0.78, 1.25) | | 1.00 (0.79, 1.27) | |  |
| Pyrethroids (insecticides) | | |  | |  | |  | |
| 3-PBA | **2.04 (1.17, 3.76)** | **2.08 (1.19, 3.88)** | | **2.01 (1.13, 3.76)** | | **2.00 (1.15, 3.69)** | |  |
| cis-DCCA | **2.52 (1.33, 5.25)** | **2.51 (1.32, 5.22)** | | **2.47 (1.28, 5.21)** | | **2.60 (1.36, 5.55)** | |  |
| trans-DCCA | **2.56 (1.18, 6.03)** | **2.55 (1.18, 5.97)** | | **2.47 (1.11, 5.93)** | | **2.67 (1.21, 6.49)** | |  |
| Fungicides | | |  | |  | |  | |
| OH-P | 1.01 (0.81, 1.22) | 1.01 (0.81, 1.22) | | 0.99 (0.79, 1.20) | | 1.00 (0.80, 1.21) | |  |
| OH-T | 1.07 (0.84, 1.34) | 1.08 (0.85, 1.36) | | 1.07 (0.84, 1.37) | | 1.05 (0.83, 1.33) | |  |
| OH-TEB | 1.03 (0.64, 1.62) | 1.03 (0.63, 1.63) | | 1.05 (0.65, 1.65) | | 1.06 (0.64, 1.70) | |  |
| Herbicides | | |  | |  | |  | |
| 2,4-D | 0.74 (0.37, 1.19) | 0.72 (0.35, 1.18) | | 0.74 (0.36, 1.19) | | 0.73 (0.37, 1.18) | |  |
| Annual EIS | **1.02 (1.00, 1.04)** | **1.02 (1.00, 1.04)** | | 1.02 (1.00, 1.04) | | 1.02 (1.00, 1.04) | |  |
| *Abbreviations*: BCP, 4-bromo-2-chlorophenol; 2,4-D, 2,4-dichlorophenoxyacetic acid; DCCA, 3-(2,2-dichlorovinyl)-2,2-dimethylcyclopropanecarboxylic acid; EIS, exposure intensity scores; 3-PBA, 3-phenoxybenzoic acid; OH-P, 3-hydroxy-pyrimetanil; OH-T, 5-hydroxy-thiabendazole; OH-TEB, hydroxy-tebuconazole; TCPy, 3,5,6-trichloro-2-pyridinol. Bolded results have p-value<0.05 without FDR correction. | | | | | | | | |
